# Awareness, treatment, and control of diabetes in South America: A systematic review and meta-analysis

**DOI:** 10.20945/2359-4292-2025-0026

**Published:** 2025-04-24

**Authors:** Jorge Emerson Chachaima-Mar, Alexandra Isabel Ramirez Moreno, Kenjiro Chau Ruiz, Maria Lazo-Porras

**Affiliations:** 1 Facultad de Medicina “Alberto Hurtado”, Universidad Peruana Cayetano Heredia, Lima, Peru; 2 CRONICAS Centro de Excelencia en Enfermedades Crónicas, Universidad Peruana Cayetano Heredia, Lima, Peru; 3 Division of Tropical and Humanitarian Medicine, University of Geneva, Geneva, Switzerland

**Keywords:** Diabetes mellitus, awareness, disease management, control, South America

## Abstract

Theaim of this review is to determine the proportion of awareness, treatment, and control of diabetes in the South American region. A comprehensive search was conducted using PubMed, EMBASE, SCOPUS, and LILACS databases from January 1, 2014, to March 23, 2024. We included observational, population-based studies that assessed the rates of awareness, treatment, and control of diabetes. The risk of bias was evaluated as proposed by Hoy and cols. A meta-analysis was performed using the random effects model, and heterogeneity was assessed using the I^2^ statistic. Additionally, a metaregression analysis was conducted to further explore heterogeneity. Fourteen studies met our eligibility criteria. The disease awareness meta-analysis, which included six studies, revealed that 71.7% (95% CI: 65.2%-77.8%, I^2^: 94.2%) of participants had a previous diagnosis of diabetes. The disease treatment meta-analysis, which included five studies, indicated that 64.6% (95% CI: 52.9%-75.3%, I^2^: 98.7%) of participants were receiving some form of treatment for diabetes, and 42.4% (95% CI: 36.0%-49.1%, I^2^: 96.3%) had their glycemic values within target ranges. All included studies were assessed to have a low risk of bias. In South America, the limited available evidence suggests a significant portion of individuals with diabetes remain untreated and uncontrolled. Numerous countries lack critical information on the diabetes care cascade necessary to inform health policies.

## INTRODUCTION

Diabetes mellitus (DM) increases the risk of multiple disabling complications, including chronic kidney disease, retinopathy, and neuropathy (^1^). It poses a significant threat to global health, with a steady increase in prevalence and economic burden worldwide (^2^). Notably, South America is projected to face the greatest economic burden relative to its gross domestic product by 2030 due to diabetes (^2^).

Most DM complications can be prevented by managing several metabolic parameters, particularly blood glucose levels (^3^). The management of these parameters begins with early diagnosis through screening programs, continues with easy access to treatment, and is maintained through adherence to regular health checks by trained health professionals. However, individuals with diabetes in South America encounter various barriers that may prevent them from maintaining control of the disease (^4^). These issues contribute to inequalities in diabetes care and highlight gaps that require improvement.

The cascade of care approach facilitates the identification of areas where the health system underperforms - such as diagnosis, management, or control (^5^). This approach can enable the development of effective health policies. Given the increasing burden of diabetes in the region, it is imperative for South American countries to acquire updated information regarding gaps in the cascade of diabetes care. Therefore, we conducted a systematic review of the currently available literature to assess the cascade of diabetes care in the South American region.

## MATERIALS AND METHODS

### Study design

We conducted a systematic literature review and meta-analysis, adhering to the guidelines established by the Preferred Reporting Items for Systematic Reviews and Meta-Analysis (PRISMA) (^6^). The study protocol was registered in PROSPERO (no. CRD42022340320).

### Inclusion and exclusion criteria

Our analysis focused on observational, population-based studies involving adult participants (>18 years) from South American countries. This selection was made to ensure that outcomes and findings pertinent to the general population are beneficial to policymakers. Conversely, samples derived from healthcare centers may not adequately represent the general population.

To be considered pertinent, a study had to evaluate at least one of the following outcomes: the proportion of participants with diabetes who are aware of their condition, the proportion of participants undergoing any form of diabetes medication (including insulin therapy), and the proportion of individuals with diabetes who have their condition under control. Studies analyzing DM were included, even if they did not specify “type 2 diabetes mellitus,” as 90% of diabetes cases in South America are type 2 (^7^). Only studies published in Spanish, English, and Portuguese were considered.

Our exclusion criteria comprised: 1) editorials, case-control, and experimental studies; 2) studies focused on specific populations (*e.g.*, pregnant women or exclusively type 1 DM patients); and 3) studies where the sample was sourced from hospitals or health centers.

### Search strategies and sources of information

Our search strategy encompassed Medline (via Ovid), EMBASE, SCOPUS, and LILACS, targeting studies published between January 1, 2014, and March 23, 2024. Both free terms (*e.g.*, “diagnosed”, “aware”, “medication”) and standardized terminology (*e.g.*, MESH) were utilized. The complete search strategy is detailed in **Supplementary Material 1**. We also reviewed references from studies deemed relevant to our analysis (^8^,^9^). A 10-year restriction was applied, as health policies necessitate updated data on diabetes, and we believe this time frame provides ample support for our analysis.

The search results from electronic databases were imported into Mendeley, where duplicates were eliminated. Subsequently, the data were uploaded into Rayyan QCRI software, a web platform utilized for the screening process (^10^). During the initial phase of selection, studies were assessed based on titles and abstracts, followed by a comprehensive full-text review of articles selected in the previous phase. Both phases were independently conducted by two authors, and the results were then compared; discrepancies at any stage were resolved by a third author.

### Extraction and management of information

Data were extracted from articles using a standardized Microsoft Excel sheet in duplicate. Extracted information included: (i) general information: first author, country, corresponding author’s name, year of publication, study design; (ii) participants: number of participants included in the analysis, age range, mean age, number, and proportion of patients by gender and population group (urban or rural); (iii) methods: diagnostic criteria for diabetes and cut-off values used for diagnosis (*e.g.*, glycated hemoglobin (HbA1c), fasting glucose, etc.), as well as the definitions employed for the outcomes; (iv) results: number and proportion of participants for each outcome, further stratified by gender and population group.

### Outcomes

The definitions used in this study for diabetes management were as follows: “Diabetes awareness” was defined as being previously diagnosed with DM. “Treatment” was defined as having diabetes and receiving hypoglycemic medications (including oral antidiabetics or insulin) or reporting adherence to non-pharmacological treatment (diet and exercise). ‘Control’ was defined as participants undergoing diabetes treatment who maintain a fasting plasma glucose level between 80-130 mg/dL and a postprandial glycemia below 180 mg/dL; and/or a HbA1c <7% (^11^).

### Risk of bias

The risk of bias was evaluated independently by two researchers, with disagreements resolved by a third researcher. We employed the tool designed by Hoy and cols. (^12^), which is specifically designed for assessing bias in prevalence studies. This tool consists of nine items addressing internal and external validity. It evaluates the representativeness of the target population, the appropriateness of the sampling method, and the adequacy of the response rate. It also considers whether data collection was conducted reliably, whether the case definition was clear and consistently applied, and whether the measurement instruments were valid. Additionally, it assesses whether statistical analyses accounted for the sampling design and whether the numerator and denominator were accurately defined for prevalence calculations. Each item is scored as “Yes” or “No”, with “Yes” indicating a low risk of bias. The total score classifies studies as having a high risk (0-^3^), moderate risk (^4^-^6^), or low risk (^7^-^9^) of bias.

### Analysis

A qualitative synthesis was conducted by summarizing the most important findings of the included studies according to our results and countries. The statistical analysis was performed using STATA v. 17. Heterogeneity was assessed both by comparing study characteristics and statistically by calculating I^2^ (^13^). According to the I^2^ value, heterogeneity can be classified as follows: 0-40% may not be important; 30%-60% may represent moderate heterogeneity; 50%-90% may represent substantial heterogeneity; and 75%-100% considerable heterogeneity. We conducted a meta-analysis using the random-effects model through the DerSimonian and Laird method as high heterogeneity was anticipated. Meta-regression models were also fitted to estimate the effect of study heterogeneity and assess the influence of publication year on awareness, treatment, and control rates. Additionally, we performed a sensitivity analysis for the control proportion to calculate an estimate utilizing only the recommended control parameter (i.e., HbA1c). We did not analyze publication bias due to the limited number of studies available. Finally, we also present the geographical distribution of our outcomes using a map sourced from https://yourfreetemplates.com/, licensed under the Creative Commons Attribution-No-Derivatives 4.0 International (CC BY-ND 4.0).

## RESULTS

**Figure 1:**
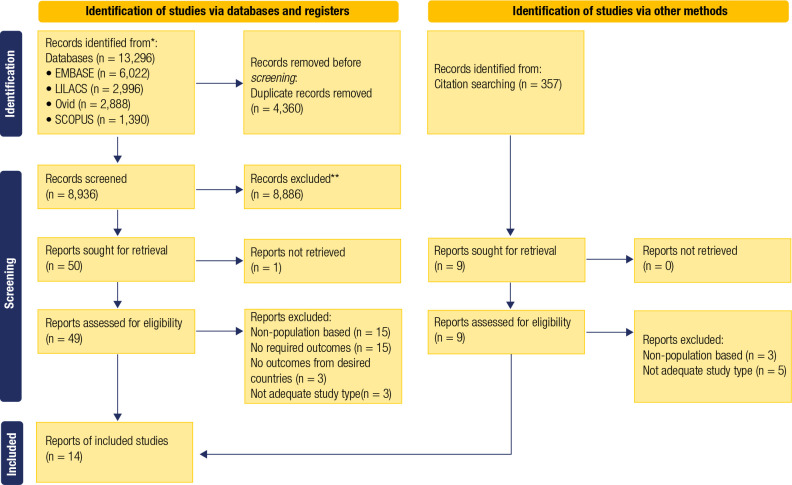
PRISMA flow diagram of study selection.

Our search encompassed 13,296 titles and abstracts, from which 4,360 duplicates were removed and 9,234 studies were excluded. We sought to retrieve 59 articles for the full-text phase. Of these, one study was not retrieved, and 44 articles were excluded during the full-text phase; the reasons are detailed in **Figure 1**, which presents the study selection process. Lastly, we included 14 studies in the review.

### Characteristics of the included studies

The characteristics of the included studies are summarized in **Supplementary Material 1**. Of the studies included, eight were conducted in Brazil (^14^-^21^), one in Colombia (^22^), one in Peru (^23^), one in Venezuela (^24^), one in Argentina (^25^), one in Chile (^26^), and one was multinational, encompassing data from Argentina, Uruguay, and Chile (^27^) **(Table 1)**. All studies were cross-sectional (^14^-^20^,^22^-^25^,^27^), with half having a national scope (^15^,^17^,^18^,^21^,^22^,^25^,^26^).

**Table 1:** Awareness, treatment, and control of DM disease in South America

Author, Year	Country	Awareness (95% IC)	Treatment (95% CI)	Control (95% CI)
Albitres-Flores, 2020	Peru	58 (51.1-65.9)	-	-
Dos Santos, 2020	Brazil	77 (73.2-80.7)	-	-
Nieto-Martinez, 2017	Venezuela	48.2 (38.2-57.4)	-	-
Fontanelli, 2017	Brazil	76.8 (66.2-85.4)	-	-
Irazola, 2017	Argentina	64.5 (58.1-70.8)	50 (43.5-56.5)	27.5 (21.6-33.4)
Irazola, 2017	Argentina	78.9 (72.6-85.2)	55.1 (47.7-62.5)	47.1 (39.7-54.5)
Irazola, 2017	Chile	81 (76.5-85.6)	63.9 (58.2-69.7)	46.7 (40.7-52.7)
Irazola, 2017	Uruguay	85.2 (80.4-90.1)	51.9 (45.5-58.3)	53.3 (46.9-59.7)
Stopa, 2018	Brazil	-	88.9	-
Monteiro, 2019	Brazil	-	80.2 (77.9-82.3)	-
Gagliardino, 2019	Argentina	-	-	48.8 (46.9-50.8)
Coutinho, 2021	Brazil	-	-	7.1 (5.8-8.6)
Machado-Duque, 2017	Colombia	-	-	53,9 (48.71-59.21)
Malta, 2019	Brazil	-	-	28.8 (25.78-33.25)
Moraes, 2020	Brazil	-	-	45.8 (43.01-48.63)
Tonaco, 2023	Brazil	62.63 (55.52-69.73)	57.63 (50.52-64.73%)	28.94 (21.84 -36.05)
Matute, 2024	Chile		60.68 (53.44 -67.92)	54.24 (47.00-61.49)

Data is expressed as a percentage ( %) unless otherwise stated.

The studies primarily utilized criteria from the American Diabetes Association (^14^,^15^,^22^-^25^,^28^), the World Health Organization (^17^,^27^), and the Brazilian Society of Diabetes (^16^,^21^). Of the 14 included studies, four exclusively assessed disease awareness (^15^,^19^,^23^,^24^), three examined only disease treatment (^18^,^20^,^26^), five focused solely on disease control (^14^,^16^,^17^,^21^,^22^,^25^,^26^), and two studies (^21^,^27^) evaluated all three outcomes, sought in this systematic review concurrently. Only one study (^14^) presented results by gender, and none reported them according to population distribution (urban vs. rural).

### Diabetes awareness

Six studies, encompassing 2,699 participants, evaluated disease awareness (^15^,^19^,^21^,^23^,^24^,^27^). These investigations utilized two distinct definitions: (i) self-reported previous knowledge of the disease at the time of the interview (^19^,^21^,^24^) and (ii) self-reported diagnosis combined with confirmatory laboratory values (^15^,^23^,^27^).

**Figure 2:**
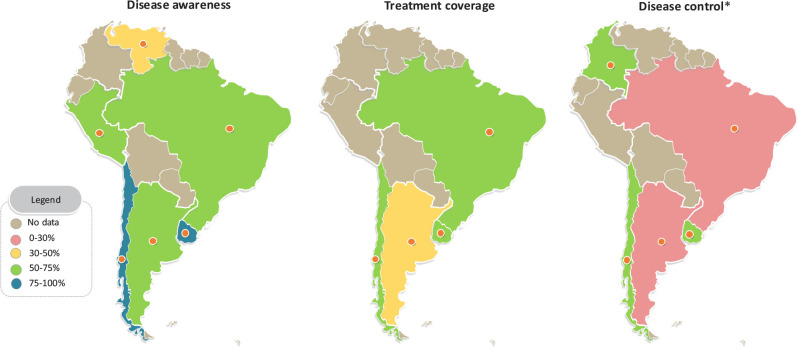
Geographical distribution of diabetes awareness, treatment, and control rates in South America.

**Figure 3:**
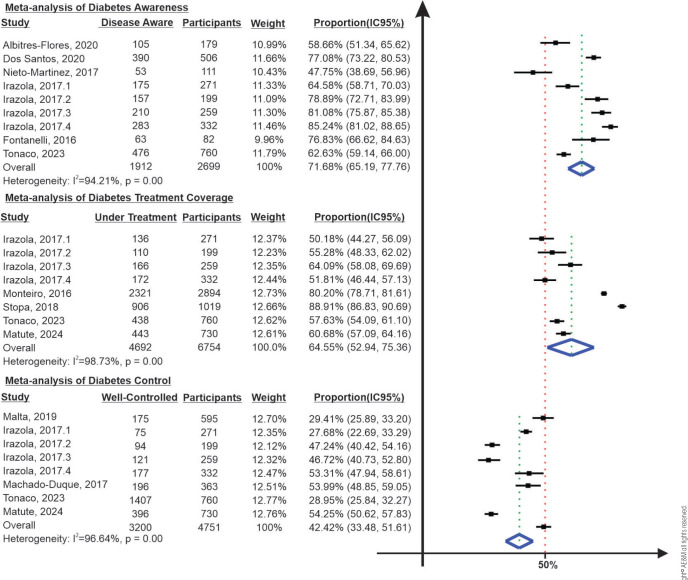
Pooled estimates of diabetes awareness, treatment, and control in South America.

Venezuela exhibited the lowest proportion of awareness (47.7%) (^24^), followed by Peru (58.7%) (^23^). Conversely, Chile and Uruguay demonstrated the highest rates of awareness, at 81.0% and 85.2%, respectively (**Figure 2**) (^27^). A meta-analysis of these six studies yielded an estimated awareness rate of 71.7% (95% CI: 65.2%-77.8%, I^2^: 94.2%) for South America (**Figure 3**).

### Diabetes treatment

Five studies, encompassing 6,754 participants, investigated the rates of diabetes treatment (^18^,^20^,^21^,^26^,^27^). Four of these studies defined treatment as administering antidiabetic medications or insulin (^18^,^21^,^26^,^27^), whereas one study did not specify its definition (^20^).

Among individuals with diabetes, Argentina reported the lowest treatment rate (50.2%), closely followed by Uruguay (51.8%) (^27^) **(Figure 2)**. In Brazil, two studies indicated treatment rates exceeding 80% (^18^,^20^), while a third study reported a treatment rate of 57.6% (^21^). A meta-analysis of these studies yielded an overall treatment rate estimate of 64.6% (95% CI: 52.9%-75.3%, I^2^: 98.7%) for South America **(Figure 3)**.

### Diabetes control

Seven studies, involving 6,169 participants, assessed diabetes control (^14^,^16^,^17^,^22^,^25^,^27^). The definition of adequate control varied across the studies. Using fasting plasma glucose <126 mg/dL as the criterion, control rates were observed to be 27.7% and 47.2% in two Argentinian cities (^27^), 53.3% in Uruguay (^27^), and between 46.7% and 54.2% in Chile (^26^,^27^). In Brazil, Malta and cols. (^17^) reported that 29.4% of individuals with diabetes had HbA1c values below 7%, whereas Moraes and cols. (^16^) found 45.8% using a stricter HbA1c target (<6.5%). In Colombia, the control rate based on HbA1c was 53.9% (^22^).

When incorporating additional parameters (blood pressure and low-density lipoprotein cholesterol), the proportion of controlled diabetes cases in Argentina was 48.8% (^25^) **(Supplementary Material 2)**. Coutinho and cols. applied the most stringent definition, including five parameters, resulting in a control rate of 7.1% in Brazil (^14^).

A meta-analysis of studies assessing only glycemic control yielded a pooled estimate of 42.4% (95% CI: 36.0-49.1, I^2^: 96.30%) for South America **(Figure 3)**. A sensitivity analysis including only studies using HbA1c as the parameter produced a pooled estimate of 39.2% (95% CI: 28.3-50.7, I^2^: 97.40%). Meta-regression models revealed no correlation between publication year and awareness, treatment, or control rates, as indicated by near-zero coefficients and confidence intervals crossing this point (results not shown).

### Risk of bias

Of the 14 studies included, all had a low risk of bias (^14^-^20^,^22^-^25^,^27^,^28^), with an average score of 9.7 out of a maximum of 10 **(Supplementary Material 4)**. Regarding external validity, all studies posed a low risk of bias, as most had national representation. Concerning internal validity, four studies were considered to have a high risk of bias because they used data collected from databases and not directly from participants (^19^,^22^,^25^,^26^).

## DISCUSSION

This systematic review, which evaluates data from countries in South America, reveals heterogeneity in DM awareness, treatment, and control rates across these populations. Additionally, diversity in terms of criteria used to define these outcomes was identified.

According to our results, only seven out of ten individuals with diabetes in South America are aware of their condition. South American countries cannot afford strategies that involve costly screening methods (*e.g.*, massive screening campaigns using blood tests); thus, low-cost auxiliary tools, such as risk scores (*e.g.*, the Finnish Diabetes Risk Score), should be encouraged in primary care guidelines to identify people at risk of diabetes (^29^). Nonetheless, regional guidelines vaguely recommend using validated risk scores without specifying them (^30^). Locally validated risk scores exist, have sufficient evidence to support their use, and should be recommended (^30^).

Peru and Venezuela exhibited the lowest levels of awareness. These two countries are characterized by difficult access to healthcare, a weak primary healthcare system, and a lack of resources in healthcare systems that do not allow for adequate detection of cases (^31^-^34^). Conversely, the highest awareness rate was found in Chile and Uruguay (^27^), both slightly surpassing the recommended proportion of 80% for this metric, according to the World Health Organization (^35^). Chile has been increasing healthcare access, with more than 95% of the population having insurance (^36^,^37^); meanwhile, Uruguay has been leading the prevention and screening of non-communicable diseases in South America by strengthening food policy and addressing cardiovascular risk factors through robust healthcare policies (^38^).

Our findings indicate that in South America, only 66.3% of individuals diagnosed with diabetes receive treatment, and 43.2% achieve glycemic control. We found Argentina to be at the lowest end of these two parameters despite having a public national program that coordinates interventions for preventing and controlling diabetes and its chronic complications. Recently, Argentina implemented a public program for the free provision of drugs at a national level (^39^). However, the public system covers only 50% of the population, while the rest - including the social security and private health system - might still encounter economic constraints in acquiring such medications (^39^).

Additionally, the recent political and economic crisis in Argentina may be hindering the improvement of such parameters. Brazil had the highest proportion of people with diabetes receiving treatment (^18^,^20^), comparable to European countries such as Switzerland (86.3%) (^40^), yet it still failed to bring half of its population under control (^16^). Brazil launched programs such as *Farmácia Popular* [Popular Pharmacy] and *Saúde Não Tem Preço* [Health Has No Price] to improve access of low-income families to diabetes medications by making them free; these initiatives could explain such high proportions of treated patients (^18^,^41^). As a result of these programs, the acquisition of diabetes medication from exclusively public pharmacies increased from 7.4% in 2013 to 18.6% in 2019, and *Farmácia Popular* remains the most common source of diabetes medications (^42^). Nonetheless, even with these efforts, poorer glycemic control is found in those using insulin or belonging to a minority group (^16^); thus, socioeconomic inequality and difficult access could account for the persistently low rate of control in this country (^21^).

When multiple cardiovascular risk factors coexist, they synergistically increase the likelihood of complications (^43^). The only study that defined control, including HbA1c, transaminases, lipids, and blood pressure, found it to be 7.1% in Brazil (^14^). This could suggest that key cardiovascular parameters, which are just as crucial as glycemia in preventing complications, are neglected by health systems in South America (^11^). Guidelines in lowand middle-income countries address comorbidities less commonly than in high-income countries (^44^). To better control all aspects of cardiovascular health in people with diabetes, South American guidelines must begin emphasizing the importance of identifying and managing comorbidities in individuals with diabetes to improve this holistic control parameter.

Our meta-regression model found no association between the year of publication and any of our three outcomes, indicating that they remained unchanged during the studied period. Recent innovative approaches and interventions have proven effective in lowand middle-income settings. These include empowering non-physician healthcare workers (*e.g.*, pharmacists or nurses) to take on more responsibilities in the management of diabetes or including the use of new technologies (*e.g.*, glucose telemonitoring or mHealth) (^45^). These strategies could provide new solutions to longstanding problems in South America: a shortage of doctors per patient and populations spread across vast distances.

## LIMITATIONS

Despite our promising findings, our study has several important limitations that should be considered. Firstly, we included only studies published in the last ten years. This allows us to evaluate more up-to-date data but might also present a fragmented picture by omitting older studies.

More than half of the studies assessing glycemic control rates employed a definition based on fasting plasma glucose, which is not the recommended metric according to diabetes guidelines (^1^). The recommended diagnostic test, HbA1c, remains expensive and inconvenient, making it challenging to implement in epidemiological studies (^1^). Point-of-care HbA1c is currently being studied to overcome these disadvantages and may hopefully be adopted in future epidemiological studies (^46^).

Lastly, despite an exhaustive literature search, we identified fewer studies than other reviews. Notably, we did not find any studies from certain countries, such as Ecuador or Paraguay. Our study maintained strict inclusion criteria to better inform policymakers, which could account for this discrepancy. The absence of data from these countries underscores a significant gap in the currently available literature regarding the diabetes care cascade in these regions. Considering that such data could also provide insights into the performance of a country’s health system (^5^), efforts should be directed toward closing this gap to better inform future healthcare policies.

In conclusions, a high proportion of individuals with diabetes mellitus in South America are unaware of their disease, do not receive treatment, and fail to achieve glycemic control. Although the studies we identified posed a low risk of bias, most South American countries lack comprehensive evidence on the proportion of individuals with diabetes who are aware, receiving treatment, or maintaining glycemic control. The deficiency of information in the diabetes care cascade necessary to guide health policies in South America is a significant concern that demands immediate attention.

## Data Availability

the data supporting the findings of this study are publicly available and can also be obtained from the corresponding author upon request.

## SUPPLEMENTARY MATERIAL

**Supplementary material 1:** Ovid (Medline), EMBASE, LILACS, SCOPUS search strategy

**OVID Search Strategy (Medline)**	
Search	Results
((exp type 2, diabetes mellitus/ or diabet*.mp. or ((“type II” or “type 2” or “Adult Onset” or “Noninsulin Dependent”) adj3 diabet*).mp. or “T2D”.mp. or NIDDM.mp.) and (exp therapeutics/or “medication”.mp. or treat$.mp. or exp diagnosis/ or “awareness”.mp. or “diagnosed”.mp. or “aware”.mp. or exp medication adherence/ or “treatment adherence”.mp. or “treatment compliance”.mp. or “control”.mp. or “uncontrolled”.mp.) and (“Argentina” or “Bolivia” or “Brazil” or “Brasil” or “Chile” or “Colombia” or “Ecuador” or “Guyana” or “Paraguay” or “Peru” or “Suriname” or “Uruguay” or “Venezuela” or “South America” or south amer$).mp.) not (exp animals/ not humans.sh.) limit 1 to yr=”2017 -Current”	2888
**LILACS search strategy**	
Search	Results
(((mh:”Diabetes Mellitus, Type 2” OR (tw:((diabet*)) OR tw:(“type 2” OR “type II” OR “Non-Insulin-Dependent” OR “Adult Onset”))) AND ((mh: diagnosis OR tw: awareness OR tw: diagnosed OR tw: aware OR tw: detected) OR (mh:therapeutics OR tw: treat$ OR tw: medication) OR (mh: “medication adherence” OR tw: “treatment compliance” OR tw: “treatment adherence” OR tw: control OR tw: uncontrolled))) AND (tw: “South America” OR “Sudamérica” OR” “Suramerica” OR “Argentina” OR “Bolivia” OR “Brasil” OR “Brazil” OR “Chile” OR “Colombia” OR “Ecuador” OR “Guyana” OR “Guiana” OR “Paraguay” OR “Peru” OR “Suriname” OR “Uruguay” OR “Venezuela”)) and not ( tw: animal$) AND ( db:(“LILACS”)) AND (year_cluster:[2017 TO 2023])	2996
**SCOPUS search strategy**	
Search	Results
TITLE-ABS-KEY ((“type 2” OR “type II” OR “Non Insulin Dependent” OR “Adult Onset” OR diabet*)) AND TITLE-ABS-KEY ((diagnosis OR aware OR unaware* OR undetected) OR (therapeutic* OR treat* OR medication) OR (“treatment adherence” OR “treatment compliance” OR “medication adherence” OR control OR uncontrolled)) AND TITLE-ABS-KEY ((“South America” OR “South America*” OR “South America*” OR “Andes” OR “Andean*” OR “Amazon*” OR “Argentina” OR “Bolivia” OR “Brazil” OR “Brazil” OR “Brazil” OR “Colombia” OR “Colombia” OR “Chile” OR “Ecuador” OR “Ecuator” OR “Guiana” OR “Guyana” OR “Paraguay” OR “Peru” OR “Suriname” OR “Uruguay” OR “Venezuela”)) AND NOT (TITLE-ABS-KEY: (animal*)) AND NOT DBCOLL ( medl ) AND ( LIMIT-TO ( PUBYEAR , 2023 ) OR LIMIT-TO ( PUBYEAR , 2022 ) OR LIMIT-TO ( PUBYEAR , 2021 ) OR LIMIT-TO ( PUBYEAR , 2020 ) OR LIMIT-TO ( PUBYEAR , 2019 ) OR LIMIT-TO ( PUBYEAR , 2018 ) OR LIMIT-TO ( PUBYEAR , 2017 ) ).	1390
**EMBASE search strategy**	
Search	Results
(exp type 2, diabetes mellitus/ or diabet*.mp. or ((“type II” or “type 2” or “Adult Onset” or “Noninsulin Dependent”) adj3 diabet*).mp. or “T2D”.mp. or NIDDM.mp.) and (exp therapeutics/ or “medication”.mp. or treat$.mp. or exp diagnosis/ or “awareness”.mp. or “diagnosed”.mp. or “aware”.mp. or exp medication adherence/ or “treatment adherence”.mp. or “treatment compliance”.mp. or “control”.mp. or “uncontrolled”.mp.) and (“Argentina” or “Bolivia” or “Brazil” or “Brasil” or “Chile” or “Colombia” or “Ecuador” or “Guyana” or “Paraguay” or “Peru” or “Suriname” or “Uruguay” or “Venezuela” or “South America” or south amer$).mp.	
limit 1 to yr=”2014 -Current”	6022

**Supplementary Material 2:** Population characteristics of the included studies.

Author, Publication Year	Level of Study	Study Design	Sample Size (N)	Age (years)
Argentina				
Gagliardino, 2019	National	Cross-sectional	2,551	Not specified
Brazil				
Coutinho, 2021	Subnational	Cross-sectional	1,418	35-74
Moraes, 2020	Subnational	Cross-sectional	1,242	35-74
Dos Santos, 2020	National	Cross-sectional	1,947	>50
Fontanelli, 2017	Community	Cross-sectional	569	>20
Stopa, 2018	Community	Cross-sectional	3,184	>20
Malta, 2019	National	Cross-sectional	8,952	>18
Monteiro, 2019	National	Cross-sectional	3,610	>18
Tonaco, 2023	National	Cross-sectional	8,435	>18
Chile				
Matute, 2024	National	Cross-sectional	5,913	>15
Colombia				
Machado-Duque, 2017	National	Cross-sectional	363	20-95
Peru				
Albitres-Flores, 2020	Community	Cross-sectional	1,500	30-69
Venezuela				
Nieto-Martinez, 2017	Community	Cross-sectional	1,334	>20
Multinational				
Irazola, 2017	Multinational (Argentina, Uruguay, Chile)	Cross-sectional	7,407	35-74

**Supplementary material 3:** Definitions of the outcomes in the included studies.

Author, year	Outcome	Definition
Coutinho, 2021	Control	HbA1c ≤7.0 % (≤53.0 mmol/mol) BP <140/90 mmHg TG <1.7 mmol/L (<150 mg/dL) LDL-C <2.6 mmol/L (< 100 mg/dL) Women: HDL-C >1.3 mmol/L (≥50 mg/dL) Men: HDL-C >1.0 mmol/L (≥40 mg/dL)
Albitres-Flores, 2020	Awareness	Self-report and FPG ≥126 mg/dL (7.0 mmol/L) or PPG at 2 h ≥200 mg/dL (11.1 mmol/L) or HbA1c ≥6.5 % (48 mmol/mol)
Dos Santos, 2020	Awareness	Self-report of diabetes and HbA1c≥ 6.5%
Machado-Duque, 2017	Control	HbA1c ≤ 7.0%
Moraes, 2020	Control	HbA1c < 6.5%
Nieto-Martinez, 2017	Awareness	Self-report
Gagliardino, 2019	Control	HbA1c ≤7% BP ≤130/80 mmHg LDL-C ≤100 mg/dL
Fontanelli, 2017	Awareness	Self-report
Stopa, 2018	Treatment	Self-report
Malta, 2019	Control	HbA1c ≤7%
Monteiro, 2019	Treatment	Self-report
Irazola, 2017	Awareness	Self-report
Irazola, 2017	Treatment	Use of antidiabetic medications
Irazola, 2017	Control	Pharmacological treatment of DM with a FPG < 126 mg/dL
Tonaco, 2023	Awareness	Self-report or use of an antidiabetic medication
Tonaco, 2023	Treatment	Use of antidiabetic medications
Tonaco, 2023	Control	HbA1c < 7%
Matute, 2024	Treatment	Under pharmacological treatment for diabetes mellitus
Matute, 2024	Control	FPG ≤ 126 mg/dL
HbA1c: glycosylated hemoglobin; SBP: Systolic blood pressure; BP: Blood pressure ; TG: triglycerides; FPG:Fasting Plasma Glucose; PPG: Postprandial Glucose		
Author, Year	Definition	
Awareness		
Albitres-Flores, 2020	Self-report and FPG ≥ 126 mg/dL (7.0 mmol/L) or PPG at 2 h ≥ 200 mg/dL (11.1 mmol/L) or HbA1c ≥ 6.5% (48 mmol/mol)	
Dos Santos, 2020	Self-report of diabetes and HbA1c ≥ 6.5%	
Nieto-Martinez, 2017	Self-report	
Irazola, 2017	Self-report	
Tonaco, 2023	Self-report or use of an antidiabetic medication	
Treatment		
Stopa, 2018	Self-report	
Monteiro, 2019	Self-report	
Irazola, 2017	Use of antidiabetic medications	
Tonaco, 2023	Use of antidiabetic medications	
Matute, 2024	Under pharmacological treatment for diabetes mellitus	
Control		
Coutinho, 2021	HbA1c ≤ 7.0% (≤ 53.0 mmol/mol); BP < 140/90 mmHg; TG < 1.7 mmol/L (< 150 mg/dL); LDL-C < 2.6 mmol/L (< 100 mg/dL); HDL-C: Women > 1.3 mmol/L, Men > 1.0 mmol/L	
Machado-Duque, 2017	HbA1c ≤ 7.0%	
Gagliardino, 2019	HbA1c ≤ 7%; BP ≤ 130/80 mmHg; LDL-C ≤ 100 mg/dL	
Moraes, 2020	HbA1c < 6.5%	
Malta, 2019	HbA1c ≤ 7%	
Irazola, 2017	Pharmacological treatment of DM with a FPG < 126 mg/dL	
Tonaco, 2023	HbA1c < 7%	
Matute, 2024	FPG ≤ 126 mg/dL	
HbA1c: glycosylated hemoglobin; SBP: Systolic blood pressure; BP: Blood pressure; TG: triglycerides; FPG:Fasting Plasma Glucose; PPG: Postprandial Glucose		

**Supplementary material 4:** Assessment of the risk of bias of the included studies with the tool proposed by Hoy et al [13].

Tool by Hoy et al. Item	Coutinho, 2021	Albitres-Flores, 2020	Dos Santos, 2020	Machado-Duque, 2017	Moraes, 2020	Nieto-Martinez, 2017	Gagliardino, 2019	Fontanelli, 2017	Stopa, 2018	Malta, 2019	Monteiro, 2019	Irazola, 2017	Tonaco, 2023	Matute, 2024
External validity														
Representativeness of the target population	LOW	HIGH	LOW	LOW	LOW	LOW	LOW	LOW	LOW	LOW	LOW	LOW	LOW	LOW
Representativeness of the sample frame	LOW	LOW	LOW	LOW	LOW	LOW	LOW	LOW	LOW	LOW	LOW	LOW	HIGH	LOW
Random sampling or census	LOW	LOW	LOW	LOW	LOW	LOW	LOW	LOW	LOW	LOW	LOW	LOW	LOW	LOW
Minimal response bias	LOW	LOW	LOW	LOW	LOW	LOW	LOW	LOW	LOW	LOW	LOW	LOW	HIGH	LOW
Internal validity														
Data were collected directly	LOW	LOW	LOW	HIGH	LOW	LOW	HIGH	HIGH	LOW	LOW	LOW	LOW	LOW	LOW
Acceptable case definition used in the study	LOW	LOW	LOW	LOW	LOW	LOW	LOW	LOW	LOW	LOW	LOW	LOW	LOW	LOW
Valid and reliable measurement	LOW	LOW	LOW	LOW	LOW	LOW	LOW	LOW	LOW	LOW	LOW	LOW	LOW	LOW
The same mode of data collection for all study subjects	LOW	LOW	LOW	LOW	LOW	LOW	LOW	LOW	LOW	LOW	LOW	LOW	LOW	LOW
Adequate duration of the prevalence period for the parameter of interest	LOW	LOW	LOW	LOW	LOW	LOW	LOW	LOW	LOW	LOW	LOW	LOW	LOW	LOW
Appropriate numerators and denominators of interest	LOW	LOW	LOW	LOW	LOW	LOW	LOW	LOW	LOW	LOW	LOW	LOW	LOW	LOW
Score	0	1	0	1	0	0	1	1	0	0	0	0	2	0
Summary of risk of bias	LOW RISK	LOW RISK	LOW RISK	LOW RISK	LOW RISK	LOW RISK	LOW RISK	LOW RISK	LOW RISK	LOW RISK	LOW RISK	LOW RISK	LOW RISK	LOW RISK

**Supplementary material 5:** PRISMA Checklist

Section and Topic	Item #	Checklist item	Location where item is reported
TITLE			
Title	1	Identify the report as a systematic review.	1
ABSTRACT			
Abstract	2	See the PRISMA 2020 for Abstracts checklist.	
INTRODUCTION			
Rationale	3	Describe the rationale for the review in the context of existing knowledge.	4
Objectives	4	Provide an explicit statement of the objective(s) or question(s) the review addresses.	4
METHODS			
Eligibility criteria	5	Specify the inclusion and exclusion criteria for the review and how studies were grouped for the syntheses.	5
Information sources	6	Specify all databases, registers, websites, organisations, reference lists and other sources searched or consulted to identify studies. Specify the date when each source was last searched or consulted.	6
Search strategy	7	Present the full search strategies for all databases, registers and websites, including any filters and limits used.	Suppl mat.
Selection process	8	Specify the methods used to decide whether a study met the inclusion criteria of the review, including how many reviewers screened each record and each report retrieved, whether they worked independently, and if applicable, details of automation tools used in the process.	6
Data collection process	9	Specify the methods used to collect data from reports, including how many reviewers collected data from each report, whether they worked independently, any processes for obtaining or confirming data from study investigators, and if applicable, details of automation tools used in the process.	7
Data items	10a	List and define all outcomes for which data were sought. Specify whether all results that were compatible with each outcome domain in each study were sought (e.g. for all measures, time points, analyses), and if not, the methods used to decide which results to collect.	7
	10b	List and define all other variables for which data were sought (e.g. participant and intervention characteristics, funding sources). Describe any assumptions made about any missing or unclear information.	7
Study risk of bias assessment	11	Specify the methods used to assess risk of bias in the included studies, including details of the tool(s) used, how many reviewers assessed each study and whether they worked independently, and if applicable, details of automation tools used in the process.	8
Effect measures	12	Specify for each outcome the effect measure(s) (e.g. risk ratio, mean difference) used in the synthesis or presentation of results.	8
Synthesis methods	13a	Describe the processes used to decide which studies were eligible for each synthesis (e.g. tabulating the study intervention characteristics and comparing against the planned groups for each synthesis (item #5)).	8
	13b	Describe any methods required to prepare the data for presentation or synthesis, such as handling of missing summary statistics, or data conversions.	8
	13c	Describe any methods used to tabulate or visually display results of individual studies and syntheses.	8
	13d	Describe any methods used to synthesize results and provide a rationale for the choice(s). If meta-analysis was performed, describe the model(s), method(s) to identify the presence and extent of statistical heterogeneity, and software package(s) used.	8
	13e	Describe any methods used to explore possible causes of heterogeneity among study results (e.g. subgroup analysis, meta-regression).	9
	13f	Describe any sensitivity analyses conducted to assess robustness of the synthesized results.	9
Reporting bias assessment	14	Describe any methods used to assess risk of bias due to missing results in a synthesis (arising from reporting biases).	8
Certainty assessment	15	Describe any methods used to assess certainty (or confidence) in the body of evidence for an outcome.	8
RESULTS			
Study selection	16a	Describe the results of the search and selection process, from the number of records identified in the search to the number of studies included in the review, ideally using a flow diagram.	9
	16b	Cite studies that might appear to meet the inclusion criteria, but which were excluded, and explain why they were excluded.	9
Study characteristics	17	Cite each included study and present its characteristics.	10
Risk of bias in studies	18	Present assessments of risk of bias for each included study.	Suppl. Mat.
Results of individual studies	19	For all outcomes, present, for each study: (a) summary statistics for each group (where appropriate) and (b) an effect estimate and its precision (e.g. confidence/credible interval), ideally using structured tables or plots.	10
Results of syntheses	20a	For each synthesis, briefly summarise the characteristics and risk of bias among contributing studies.	Suppl. Mat.
	20b	Present results of all statistical syntheses conducted. If meta-analysis was done, present for each the summary estimate and its precision (e.g. confidence/credible interval) and measures of statistical heterogeneity. If comparing groups, describe the direction of the effect.	12
	20c	Present results of all investigations of possible causes of heterogeneity among study results.	12
	20d	Present results of all sensitivity analyses conducted to assess the robustness of the synthesized results.	12
Reporting biases	21	Present assessments of risk of bias due to missing results (arising from reporting biases) for each synthesis assessed.	13
Certainty of evidence	22	Present assessments of certainty (or confidence) in the body of evidence for each outcome assessed.	12
DISCUSSION			
Discussion	23a	Provide a general interpretation of the results in the context of other evidence.	14
	23b	Discuss any limitations of the evidence included in the review.	17
	23c	Discuss any limitations of the review processes used.	17
	23d	Discuss implications of the results for practice, policy, and future research.	15
OTHER INFORMATION			
Registration and protocol	24a	Provide registration information for the review, including register name and registration number, or state that the review was not registered.	5
	24b	Indicate where the review protocol can be accessed, or state that a protocol was not prepared.	5
	24c	Describe and explain any amendments to information provided at registration or in the protocol.	5
Support	25	Describe sources of financial or non-financial support for the review, and the role of the funders or sponsors in the review.	19
Competing interests	26	Declare any competing interests of review authors.	19
Availability of data, code and other materials	27	Report which of the following are publicly available and where they can be found: template data collection forms; data extracted from included studies; data used for all analyses; analytic code; any other materials used in the review.	19

From: Page MJ, McKenzie JE, Bossuyt PM, Boutron I, Hoffmann TC, Mulrow CD, et al. The PRISMA 2020 statement: an updated guideline for reporting systematic reviews. BMJ 2021;372:n71. doi: 10.1136/bmj.n71
